# Metformin reverses multidrug resistance in human hepatocellular carcinoma Bel-7402/5-fluorouracil cells

**DOI:** 10.3892/mmr.2014.2614

**Published:** 2014-10-08

**Authors:** SUNBIN LING, YU TIAN, HAIQUAN ZHANG, KAIQI JIA, TINGTING FENG, DEGUANG SUN, ZHENMING GAO, FEI XU, ZHAOYUAN HOU, YAN LI, LIMING WANG

**Affiliations:** 1 Division of Hepatobiliary and Pancreatic Surgery, Department of Surgery, First Affiliated Hospital, Zhejiang University School of Medicine, Hangzhou, Zhejiang 310003, P.R. China; 2 Division of Hepatobiliary and Pancreatic Surgery, Department of Surgery, The Second Affiliated Hospital of Dalian Medical University, Dalian, Liaoning 116027, P.R. China; 3 Institute of Cancer Stem Cell, Dalian Medical University, Dalian, Liaoning 116044, P.R. China; 4 Department of Intergrative Medicine, Zhejiang Cancer Hospital, Hangzhou, Zhejiang 310022, P.R. China; 5 College of Basic Medical Sciences, Dalian Medical University, Dalian, Liaoning 116044, P.R. China; 6 Department of Biochemistry and Molecular Cell Biology, Shanghai Key Laboratory for Tumor Microenvironment and Inflammation, Shanghai Jiaotong University School of Medicine, Shanghai 200025, P.R. China

**Keywords:** metformin, liver neoplasms, multidrug resistance, hypoxia-inducible factor-1α, P-glycoprotein, multidrug resistance-associated protein 1

## Abstract

Metformin exhibits anti-proliferative effects in tumor cells *in vitro* and *in vivo*. The present study investigated the ability of metformin to reverse multidrug resistance (MDR) in human hepatocellular carcinoma Bel-7402/5-fluorouracil (5-Fu; Bel/Fu) cells. The synergistic anti-proliferative effect of metformin combined with 5-Fu was evaluated using a Cell Counting kit-8 assay. The variation in apoptotic rates and cell cycle distribution were evaluated using a flow cytometric assay and variations in target gene and protein expression were monitored using reverse transcription-polymerase chain reaction and western blot analysis. The results demonstrated that metformin had a synergistic anti-proliferative effect with 5-Fu in the Bel/Fu cells. The variations in the number of apoptotic cells and distribution of the cell cycle were consistent with the variability in cell viability. Metformin targeted the AMP-activated protein kinase (AMPK)/mammalian target of rapamycin (mTOR) pathway, suppressed the expression of hypoxia-inducible factor-1α (HIF-1α) and transcriptionally downregulated the expression of multidrug resistance protein 1/P-glycoprotein (P-gp) and multidrug resistance-associated protein 1 (MRP1). Collectively, these findings suggested that metformin may target the AMPK/mTOR/HIF-1α/P-gp and MRP1 pathways to reverse MDR in hepatocellular carcinoma.

## Introduction

Hepatocellular carcinoma (HCC) is the sixth most common solid tumor in the world and the third most common cause of cancer mortality (1). Surgical resection and liver transplantation surgery are the only potential curative treatments for patients with early stage tumors. However, a number of patients with advanced HCC are not suitable for surgery due to the presence of metastases (2). Systemic chemotherapy is the main treatment method for patients with advanced HCC. However, traditional chemotherapy treatments provide little efficacy in patients with advanced HCC and often provide no benefit to survival rate (3). Recently, agents targeting certain key pathways have provided new methods for the treatment of HCC. For instance, 5-fluorouracil (5-Fu), an inhibitor of thymidylate synthase, was reported as one of the first chemotherapeutic agents assessed for the treatment of HCC with a response rate of ~10% (4). Therefore, novel pharmacological strategies for the treatment of HCC are urgently required.

Multidrug resistance (MDR), including intrinsic or acquired MDR, is a multi-factorial phenomenon and the major cause of chemotherapeutic failure during cancer treatment. MDR markedly restrains the management of clinical HCC chemotherapy. MDR effectors include the transporter glycoproteins P-glycoprotein (P-gp) and multidrug resistance-associated protein 1 (MRP1), whose overexpression is generally considered to be the underlying mechanism responsible for the MDR of tumor cells.

Metformin is a commonly used biguanide antidiabetic drug that has anti-proliferative properties and its use in diabetic patients has been linked to a reduction in the incidence of cancer (5). Metformin activates AMP-activated protein kinase (AMPK), most likely by interfering with mitochondrial respiratory complex I (6). Activation of AMPK regulates the growth of tumor cells through inhibition of the mammalian target of rapamycin (mTOR) pathway. This pathway is generally involved in the control of translation initiation and protein synthesis (7).

The mTOR pathway is aberrantly activated in HCC (8). Inhibition of mTOR has been demonstrated to suppress liver tumor growth and metastasis (9,10). Several clinical and preclinical studies have demonstrated that the mTOR inhibitor rapamycin and its analogues can be used to treat various types of solid tumor, including esophageal squamous cell carcinoma (11), lung cancer (12), renal cell carcinoma (13) and prostate cancer (14). Therefore, the mTOR pathway may be a prospective target for the treatment of HCC. Furthermore, as hypoxia has well-documented effects on cancer malignancy and resistance to chemotherapy (15), hypoxia inducible factor 1α (HIF-1α), a downstream protein of mTORC1 that regulates the expression of P-gp and MRP1, has emerged as an attractive target for cancer therapy (16).

Metformin is a potential anti-cancer agent targeting the mTOR/HIF-1α pathway. In the present study, the role of metformin as a chemosensitizer was investigated. The Bel-7402 derived multidrug-resistant cell line Bel/Fu, was used as an MDR model.

## Materials and methods

### Cell culture

The present study was approved by the Second Affiliated Hospital of Dalian Medical University Research Ethics Committee (Dalian, China). The human hepatocellular carcinoma MDR cell line Bel-7402/5-Fu (Bel/Fu) was obtained from KeyGen Biotech Co., Ltd. (Nanjing, China). The cell line was cultured in RPMI-1640 medium (Gibco-BRL, Carlsbad, CA, USA) supplemented with 10% fetal bovine serum (FBS; Gibco-BRL), 100 μg/ml penicillin and 100 μg/ml streptomycin (Invitrogen Life Technologies, Carlsbad, CA, USA) at 37°C in a 5% CO_2_ atmosphere. The MDR Bel/Fu cells were maintained in medium containing 20 μg/ml 5-Fu.

### Reagents

Metformin (cat no. D150959-5G) and 5-Fu (cat no. F6627-1G) were purchased from Sigma-Aldrich (St. Louis, MO, USA). The Cell Counting kit-8 (CCK-8; cat no. KGA317), Annexin V-fluorescein isothiocyanate (FITC) Apoptosis Detection kit (cat no. KGA108) and the Cell Cycle Detection kit (cat no. KGA512) were purchased from KeyGen Biotech Co., Ltd.

### Antibodies

The following polyclonal rabbit anti-human antibodies were used for western blot analysis: HIF-1α (N-term; cat no. AP4776a; diluted 1:1,000), active caspase-3 (cat no. AJ1131b; diluted 1:1,000), B-cell lymphoma 2 (Bcl-2; cat. no AJ1082a; diluted 1:1,000), cyclin-dependent kinase 4 (CDK4; cat no. AP7520b; diluted 1:1,000) and cyclin D1 (cat no. AP2612c, diluted 1:1,000) obtained from Abgent (San Diego, CA, USA); AMPKα (Ab-172; cat no. B0003, diluted 1:500), phosphorylated AMPKα (phospho-Thr172; cat no. A0003; diluted 1:500), mTOR (cat no. B7156; diluted 1:500) and phosphorylated mTOR (phospho-Ser2448; cat no. A7156; diluted 1:500) purchased from Assay Biotech Co., Inc. (Sunnyvale, CA, USA); P-gp (cat no. PA5-28801; diluted 1:500) and MRP1 (cat no. PA5-30594; diluted 1:500) obtained from Thermo Fisher Scientific Inc. (Rockford, IL, USA). The mouse anti-human monoclonal β-actin antibody (cat no. sc-47778, diluted 1:1,000) was purchased from Santa Cruz Biotechnology, Inc. (Santa Cruz, CA, USA). The polyclonal goat anti-rabbit and polyclonal goat anti-mouse IgG, peroxidase-conjugated secondary antibodies (cat nos. 31460 and 31430; diluted 1:10,000) were purchased from Thermo-Pierce (Rockford, IL, USA).

### Cell viability assay

Cell viability was determined using the CCK-8 assay according to the manufacturer’s instructions. Cells (5×10^3^) were seeded into each well of a 96-well plate and cultured in 100 μl RPMI-1640 medium supplemented with 10% FBS, 100 μg/ml penicillin and 100 μg/ml streptomycin. After 24 h, single (100 μg/ml 5-Fu or 10 mM Met) or combined agents (100 μg/ml 5-Fu plus 10 mM Met) were added into the culture medium. The cells were incubated at 37°C for 24, 48 or 72 h, following which the medium was exchanged for 100 μl RPMI-1640 medium and 10 μl CCK-8 reagent. The cells were incubated for 2 h at 37°C. Finally, the optical density was measured using an EnSpire™ 2300 Multilabel Reader (PerkinElmer, Inc., Waltham, MA, USA) at 450 nm. Five replicates were prepared for each condition. The mean values were calculated and growth curves were produced.

### Western blot analysis

Following the different treatments, the cells were harvested and lysed in radioimmunoprecipitation assay buffer (cat no. KGP702; KeyGen Biotech Co., Ltd.) supplemented with 1 mM phenylmethylsulfonyl fluoride (cat no. KGP610; KeyGen Biotech Co., Ltd.) and 1 mM phosphatase inhibitor cocktail (cat no. KGP602; KeyGen Biotech Co., Ltd.). The mixture was centrifuged at 12,000 × g for 20 min and the supernatant was collected. The protein concentration was determined using a BCA assay kit (cat no. KGPBCA; KeyGen Biotech Co., Ltd.) and each sample contained 30 μg protein per 10 μl. The protein samples were mixed with loading buffer (cat no. KGP101; KeyGen Biotech Co., Ltd.) and the proteins were separated using 6 or 10% sodium dodecyl sulfate-polyacrylamide gel electrophoresis and transferred onto polyvinylidene difluoride membranes (Bio-Rad, Hercules, CA, USA). Following soaking in a blocking buffer (TBS with 5% nonfat dry milk) for 2 h, the blots were incubated at 4°C overnight with the primary antibody and subsequently incubated at 37°C for 1 h with the horseradish peroxidase-conjugated secondary antibody. The bands were visualized using chemiluminescence and images were captured using a ChemiDoc XRS imaging system (Bio-Rad). These were analyzed using Image Lab software (Bio-Rad).

### Reverse transcription-polymerase chain reaction (RT-PCR)

Total RNA was isolated using an RNAiso™ Plus kit (Takara Bio, Inc., Shiga, Japan). Total RNA (500 ng) was reverse-transcribed into first-strand cDNA using a Takara RNA PCR kit (AMV Ver. 3.0; Takara Bio, Inc.) according to the manufacturer’s instructions. The following primers were used for amplification: multidrug resistance 1 (MDR1), forward 5′-CCCATCATTGCAATAGCAGG-3′ and reverse 5′-GTT CAAACTTCTGCTCCTGA-3′; MRP1, forward 5′-TGAAGG ACTTCGTGTCAGCC-3′ and reverse 5′-GTCCATGAT GGTGTTGAGCC-3′; β-actin, forward 5′-GCATGGAGTCCT GTGGCAT-3′ and reverse 5′-CTAGAAGCATTTGCG GTGG-3′. The PCR reactions were subjected to the following amplification conditions: denaturation at 94°C for 30 sec, annealing (53°C for MDR1, 57°C for MRP1 and 58°C for β-actin) for 30 sec and extension at 72°C for 30 sec. MDR1 was incubated for 32 cycles, MRP1 for 25 cycles and β-actin for 30 cycles. The PCR products were analyzed by 2% gel electrophoresis.

### Flow cytometric analysis

To evaluate the effects of single or combined agents on cell cycle arrest and the induction of apoptosis, the cells were examined using an Annexin V-FITC Apoptosis Detection kit and a Cell Cycle Detection kit, according to the manufacturer’s instructions. Bel/Fu cells were seeded into 6-well plates (1×10^5^ and 2×10^5^ cells per dish, respectively). For cell cycle analysis, following the treatment with single or combined agents for 48 h, a total of 1×10^6^ cells were pelleted by centrifugation at 1,000 × g for 5 min and washed twice with PBS. The cell pellets were then resuspended in 500 μl ice-cold 70% ethanol and incubated at 4°C overnight. The fixed cells were centrifuged at 1,000 × g for 5 min and the pellets were washed with PBS. Following incubation with 100 μl RNase A (10 μg/ml) at 37°C for 30 min in the dark, the cells were resuspended in 400 μl propidium iodide (PI; 50 μg/ml) and placed in the dark at 4°C for 30 min. The stained cells were analyzed using an Accuri C6 flow cytometer (Accuri Cytometers, Inc., Ann Arbor, MI, USA). For analysis of apoptosis, the cells were trypsinized, washed with cold PBS and suspended in PBS. Subsequently, the cells were stained using Annexin V-FITC reaction reagent (10 μl Annexin V-FITC, 5 μl PI) at 37°C in the dark for 30 min. The stained cells were analyzed using an Accuri C6 flow cytometer (Accuri Cytometers, Inc.).

### Statistical analysis

SPSS 13.0 statistical software was used for statistical analysis (SPSS, Inc., Chicago, IL, USA). Values are presented as the mean ± standard deviation. Statistical analyses were performed using Student’s t-test and the analysis of multiple groups was performed using analysis of variance, followed by the Student-Newman-Keuls post hoc test.

## Results

### Metformin has a synergistic anti-proliferative effect with 5-Fu in Bel/Fu cells

**Figure 1:**
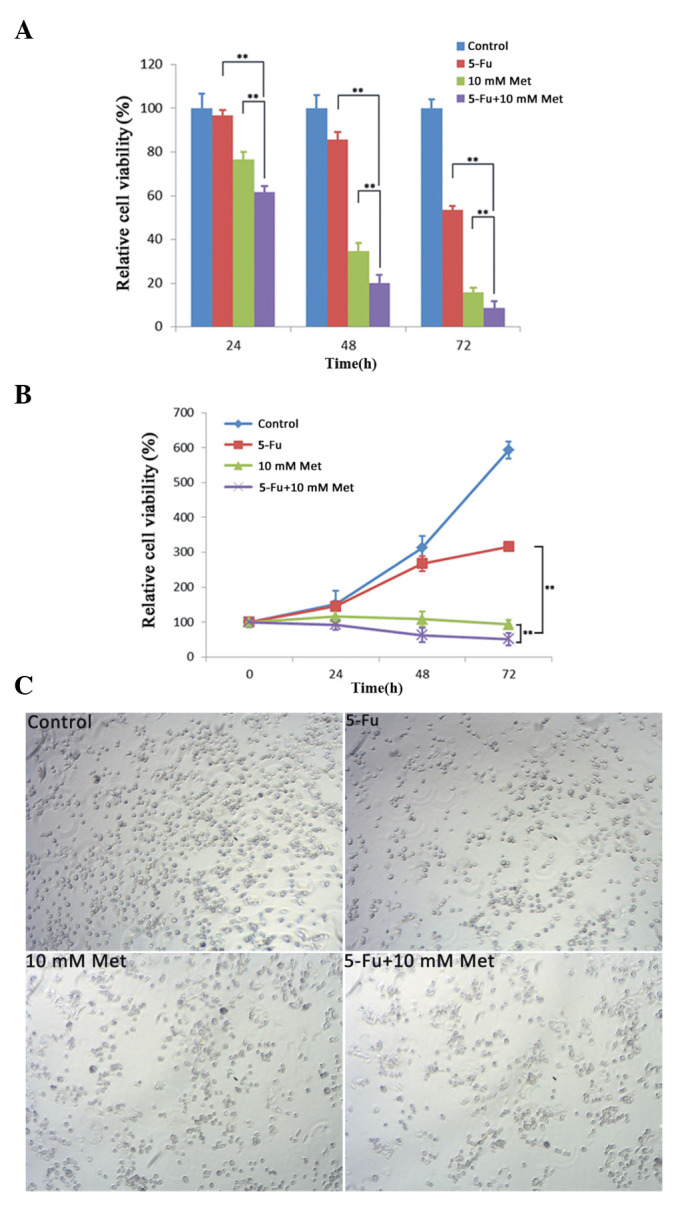
Met has a synergistic anti-proliferative effect with 5-Fu in Bel/Fu cells. (A and B) Bel/Fu cells were singly or jointly treated with 100 μg/ml 5-Fu or 10 mM met. A Cell Counting kit-8 assay was performed to determine cell viability (^**^P<0.01, compared with the 5-Fu and Met). (C) Morphology of the Bel/Fu cells following treatment with Met for 48 h (magnification, ×10). 5-Fu, 5-fluorouracil; Met, metformin.

CCK-8 analysis was used to assess the effect of 5-Fu and metformin on the proliferation of Bel/Fu cells. As shown in Fig. 1A and B, the course of proliferation was observed for 3 days after the addition of the chemotherapeutic agents. All groups exhibited a time-dependent inhibition of cell proliferation. Following treatment with 100 μg/ml 5-Fu, 10 mM metformin or a combination of the agents for 48 h, the cell viability rates were 85.58±3.76, 34.62±3.65 and 20.13±3.60%, respectively. The combination of the two agents further decreased the proliferation rate of the Bel/Fu cells compared with application of a single drug. Microscopic examination demonstrated a significant decrease in cell density, which correlated with the cell viability results. In conclusion, metformin had a synergistic anti-proliferative effect with 5-Fu in the Bel/Fu cells.

### Combined treatment with 5-Fu and metformin promotes apoptosis and induces cell cycle arrest in Bel/Fu cells

**Figure 2:**
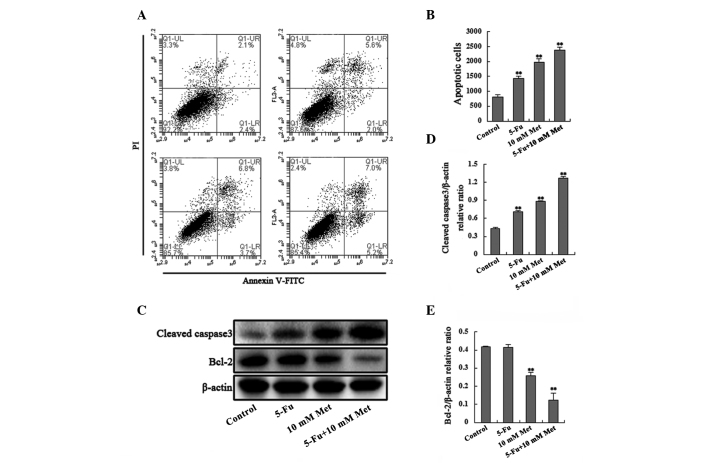
Combined treatment with 5-Fu and Met promotes apoptosis in Bel-7402/5-Fu cells. (A) Following single or combined treatment with 100 μg/ml 5-Fu and 10 mM Met, the apoptotic cells were detected by Annexin V-PI dual staining. The total cells in Q1-LR and Q1-UR quadrants were regarded as apoptotic cells (early apoptotic and late apoptotic/necrotic cells, respectively). (B) Percentages of early apoptotic and late apoptotic/necrotic cells are shown in the bar graph. (^**^P<0.01) (C) Cleaved caspase-3 and Bcl-2 levels were monitored using western blot analysis. (D and E) Band intensities were quantified using Image Lab 5.0 software and were normalized to β-actin (^**^P<0.01, compared with the control). 5-Fu, 5-fluorouracil; Bcl-2, B-cell lymphoma 2; Met, metformin; PI, propidium iodide; FITC, fluorescein isothiocyanate.

**Figure 3:**
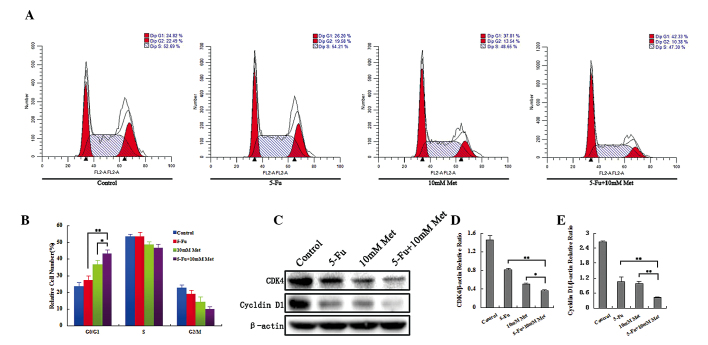
Combined treatment with 5-Fu and Met induces cell cycle arrest in Bel/Fu cells. (A) Following single or combined treatment with 100 μg/ml 5-Fu and 10 mM Met, Bel/Fu cells were stained with propidium iodide. The cell cycle distribution was measured using flow cytometric analysis. (B) Percentages of cells in each phase of the cell cycle are shown in the bar graph. (^*^P<0.05, ^**^P<0.01, the combined treatment group, compared with the single agent treatment group) (C) Levels of cyclin D1 and CDK4 were monitored using western blot analysis. (D and E) Band intensities were quantified using Image Lab 5.0 software and were normalized to β-actin. (^*^P<0.05, ^**^P<0.01, the combined treatment group, compared with the single agent treatment group). 5-Fu, 5-fluorouracil; Bcl-2, B-cell lymphoma 2; Met, metformin; CDK4, cyclin-dependent kinase 4; Bel/Fu, Bel-7402/5-Fu.

To determine whether the drug application-induced decrease in cell viability was accompanied by cell cycle arrest or apoptosis, cell cycle progression and induction of apoptosis were analyzed. The number of apoptotic cells and the percentages of cells in each phase of the cell cycle were examined using flow cytometry after 48 h of incubation with the agents (100 μg/ml 5-Fu, 10 mM Met, or 100 μg/ml 5-Fu plus 10 mM Met). A significant increase in the number of apoptotic cells and cells undergoing G0/G1 cell cycle arrest was observed in the Bel/Fu cells treated with the combined agents compared with the cells treated with a single agent (Figs. 2A, 2B, 3A and 3B). The Bel/Fu cells were cultured in the presence of the vehicle, 100 μg/ml 5-Fu, 10 mM metformin or a combination of 5-Fu and metformin for 48 h. The total percentage of apoptotic cells, including early apoptotic and late apoptotic/necrotic cells, was 4.5±0.58, 7.6±0.74, 10.5±1.21 and 12.2±0.71%, respectively. In addition, the percentage of cells in the G0/G1 phase was 24.52±2.82% for the vehicle control cells, 26.92±3.23% for the 5-Fu cells, 37.12±3.21% for the metformin cells and 43.33±3.18% for the combined treatment.

Furthermore, to confirm that the agents induced apoptosis and G0/G1 cell cycle arrest in the Bel/Fu cells, the levels of cleaved caspase-3, Bcl-2, cyclin D1 and CDK4 were monitored using western blot analysis in the Bel/Fu cells (Fig. 2C–E and Fig. 3C–E). Activation of the pro-apoptotic caspase-3 and downregulation of the apoptosis suppressor Bcl-2 were associated with agent-induced apoptosis. Cyclin D1 and CDK4, which are responsible for the transition from G0/G1 phase to the S phase, were downregulated following combined or single agent treatment. Thus, these results suggested that metformin promoted apoptosis and induced G0/G1 cell cycle arrest in the Bel/Fu cells.

### Metformin treatment results in increased AMPK phosphorylation and decreased mTOR phosphorylation in a dose-dependent manner

**Figure 4:**
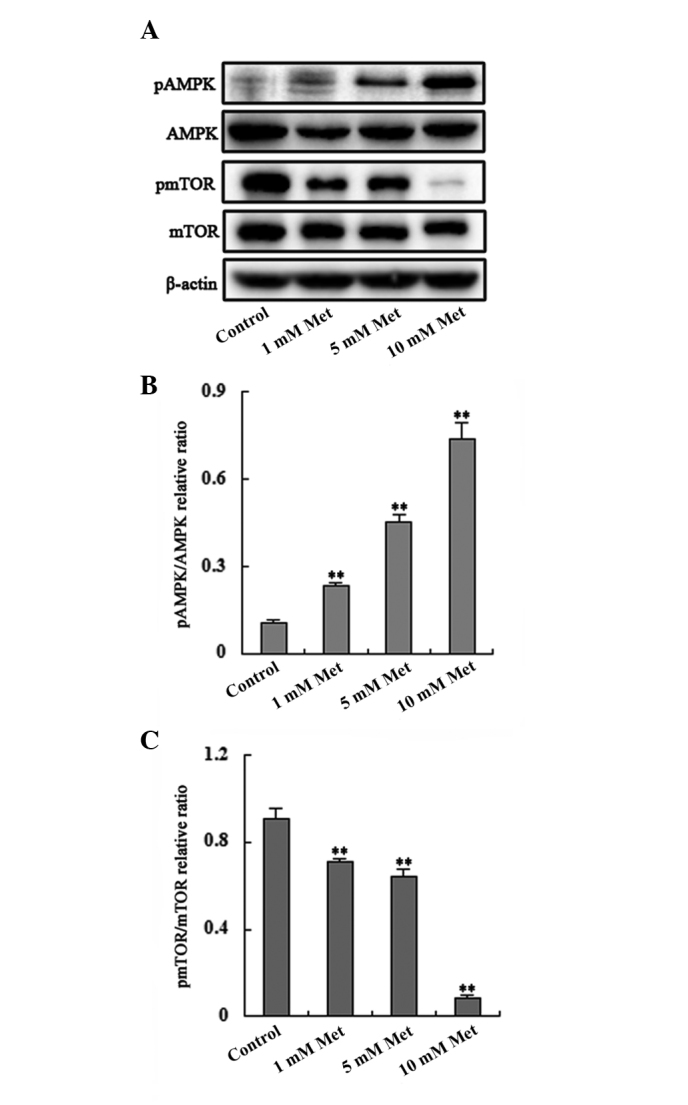
Met treatment increases phosphorylation of AMPK and decreases phosphorylation of mTOR. (A) Phosphorylation of AMPK was upregulated and phosphorylation of mTOR was downregulated in a dose-dependent manner. (B and C) Band intensities were quantified using Image Lab 5.0 software and were normalized to β-actin (^**^P<0.01, compared with the control). AMPK, AMP-activated protein kinase; mTOR, mammalian target of rapamycin; pAMPK, phosphorylated AMP-activated protein kinase; pmTOR, phosphorylated mammalian target of rapamycin; Met, metformin.

To evaluate the specific effect of metformin on the AMPK/mTOR pathway, which is widely considered to be the most common target of metformin, western blot analysis was used to evaluate the AMPK/mTOR pathway in Bel/Fu cells. Metformin treatment resulted in enhanced AMPK phosphorylation and reduced mTOR phosphorylation in a dose-dependent manner (Fig. 4).

### Metformin treatment results in decreased HIF-1α, MDR1 and MRP1 expression in Bel-Fu cells

**Figure 5:**
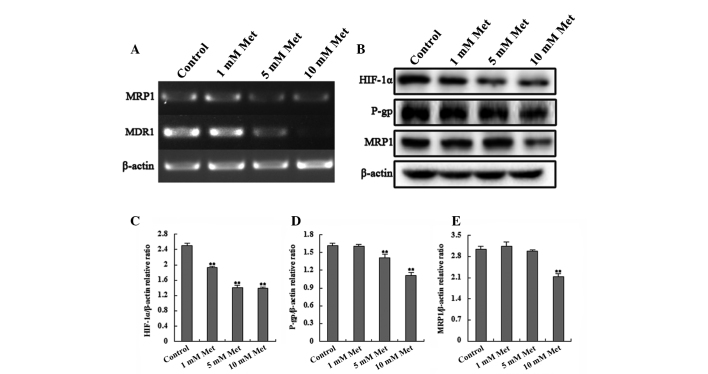
Met affects the expression of HIF-1α, MDR1/P-gp and MRP1. (A) Reverse transcription-polymerase chain reaction analysis of MDR1 and MRP1 expression in the different treatment groups. (B) Western blot analysis of HIF-1α, MDR1 and MRP1 in the different treatment groups. (C–E) Relative density of the western blotting bands demonstrated that levels of HIF-1α, P-gp and MRP1 decreased with increasing concentrations of Met in a dose-dependent manner, with a statistically significant difference between the treatments and the controls (^**^P <0.01, compared with the control). P-gp and MRP1 protein band densities were normalized to their corresponding β-actin bands. (^**^P<0.01, compared with the control). HIF-1α, hypoxia-inducible factor-1α; MDR1, multidrug resistance-associated protein 1; P-gp, P-glycoprotein; MRP1, multidrug resistance protein 1; Met, metformin.

HIF-1α is a downstream protein of the mTOR pathway that transcriptionally regulates the expression of P-gp/MDR1 and MRP1, which are associated with MDR. Thus, the present study examined the expression of these genes in Bel/Fu cells using RT-PCR and western blot analysis in order to determine the effects of metformin on these genes. The results demonstrated that the expression of HIF-1α, MDR1 and MRP1 in the Bel/Fu cells decreased significantly with increasing concentrations of metformin (Fig. 5). HIF-1α may transcriptionally regulate the expression of P-gp/MDR1 and MRP1.

## Discussion

HCC is the sixth most common solid tumor in the world and the third leading cause of cancer mortality worldwide (1). MDR is a multifactorial phenomenon and the major obstacle in the successful and effective chemotherapeutic treatment of cancer. HCC can easily acquire MDR, which leads to unsatisfactory effects during chemotherapy treatment (17). Therefore, traditional chemotherapy treatments achieve little efficacy in patients with advanced HCC and are often no benefit to survival rates (3). Thus, approaches to reduce the MDR properties of cancer cells may enhance the efficacy of chemotherapeutic agents in the treatment of HCC.

Metformin is a representative therapeutic agent for type 2 diabetes mellitus. Previous studies have reported that metformin activates AMPK (18) and inhibits hepatic cancer cell growth (19). However, the effect of metformin on the MDR of HCC cells remains to be elucidated. The present study demonstrated that metformin reversed the MDR of Bel/Fu cells, which were used as an MDR model, by targeting the AMPK/mTOR/HIF-1α pathway.

To evaluate the effect of metformin on the Bel/Fu cells, the cell viability of cells treated with vehicle controls (100 μg/ml 5-Fu, 10 mM metformin and combined treatment of 100 μg/ml 5-Fu and 10 mM metformin) was examined. The cell viability was more significantly inhibited in the combination group than in the single agent groups and the cell viability inhibition rate was also time dependent. The combination of the two agents decreased the proliferation rate of the Bel/Fu cells compared with the single drug treatments. Additionally, the apoptotic rate increased consistently with the decrease in cell viability. These results indicated that metformin may reverse the MDR of Bel/Fu cells.

The sensitivity of cells to chemotherapy may be evaluated by examining the cell cycle distribution and the present study analyzed this in the different treatment groups. As mentioned above, no significant different was identified between the 5-Fu group and the control group. However, the proportion of cells in the G1 phase was markedly increased in the 10 mM metformin group and in the combination group, while the proportion of cells in the S, G2 and M phases decreased concordantly. Therefore, the present study demonstrated that the expression of cyclin D1 and CDK4, which facilitate transition from the G1 to the S phase during cell proliferation, were decreased in the 5-Fu, 10 mM metformin and combination treatment groups compared with the control group. As cell apoptosis is another important indicator used to measure the chemotherapeutic sensitivity of cells, the apoptotic rate of each group was analyzed using flow cytometry. The apoptotic rate of the 5-Fu + 10 mM metformin group was significantly higher than the other groups. Coincidently, the expression of cleaved caspase-3 increased and the expression of Bcl-2 decreased in the 5-Fu, 10 mM metformin and combination treatment groups compared with the control group. These findings confirmed that metformin reversed the MDR of the Bel/Fu cells.

Previous studies have demonstrated that P-gp and MRP1, which act as energy-dependent drug efflux pumps, are important in MDR (20). According to previous studies, mTORI decreases the translation of HIF-1α (21). Overexpression of HIF-1α significantly upregulates the expression of P-gp and MRP1, indicating that HIF-1α may confer hypoxia-induced drug resistance by reducing intracellular drug accumulation (22,23). However, metformin inhibits the phosphorylation of mTOR by activating AMPK (23). Therefore, the present study also evaluated the expression of these proteins using western blot analysis. The activation of AMPK increased with increasing concentrations of metformin. By contrast, the phosphorylation of mTOR was inhibited, leading to a subsequent decrease in HIF-1α. Coincidently, the expression of P-gp and MRP1 decreased in the metformin-treated cells in a dose-dependent manner at the mRNA and protein levels; P-gp is a translational product of the MDR1 gene.

Previous studies have suggested that metformin may improve oxygenation and suppress the accumulation of HIF-1α in diseases associated with tumors or diabetes through the activation of the AMPK/mTOR pathway and the repression of oxygen consumption (24–27). HIF-1, a basic helix-loop-helix transcription factor, is important in regulating the transcription of various target genes in response to hypoxia (28). HIF-1α is an oxygen-regulated subunit that mediates the essential function of HIF-1. Previous studies have demonstrated that the overexpression of HIF-1α may contribute to the pathogenesis of tumor resistance to chemotherapy (29,30). P-gp and MRP1 are regarded as energy-dependent membrane efflux pumps and are widely considered to be transcriptionally regulated by HIF-1α in multiple types of human tumor (22,31–33). Consequently, the results of the present study implied that metformin reversed the MDR of Bel/Fu cells by targeting the AMPK/mTOR/HIF-1α/P-gp and MRP1 signaling pathways. Therefore, metformin may be important in combination chemotherapy treatments for HCC and other malignances.

In conclusion, the present study revealed the role of metformin, an inexpensive and widely used antidiabetic drug without severe adverse effects, in reversing the MDR of HCC cells. Metformin may target the AMPK/mTOR/HIF-1α/P-gp and MRP1 pathways, resulting in sensitization of the cells to combination chemotherapies with other agents for the treatment of HCC. Future studies may focus on the chemotherapy sensitization effect of metformin with other chemotherapeutics *in vivo* and on the elucidation of the precise mechanism underlying the cytotoxic and MDR reversal effects of metformin in HCC.
