# Metformin reverses multidrug resistance in human hepatocellular carcinoma Bel-7402/5-fluorouracil cells

**DOI:** 10.3892/mmr.2022.12691

**Published:** 2022-03-21

**Authors:** Sunbin Ling, Yu Tian, Haiquan Zhang, Kaiqi Jia, Tingting Feng, Deguang Sun, Zhenming Gao, Fei Xu, Zhaoyuan Hou, Yan Li, Liming Wang

Mol Med Rep 10: 2891–2897, 2014; DOI: 10.3892/mmr.2014.2614

Subsequently to the publication of the above paper, the authors have reviewed its content and the primary data, and have realized that the western blots selected to show the β-actin experiments featured in Fig. 4A and Fig. 3C were the same blot, albeit with a different exposure time. The control blots correctly presented for Fig. 3C were inadvertently copied into Fig. 4A owing to an error made during the figure compilation process.

The revised version of Fig. 4, containing the correct β-actin blots for Fig. 4A, is shown below. Note that this error did not significantly affect the results or the conclusions reported in this paper, and all the authors agree to this Corrigendum. The authors thank the Editor of *Molecular Medicine Reports* for allowing them the opportunity to publish this corrigendum, and apologize to the readership for any inconvenience caused.

## Figures and Tables

**Figure 4.**
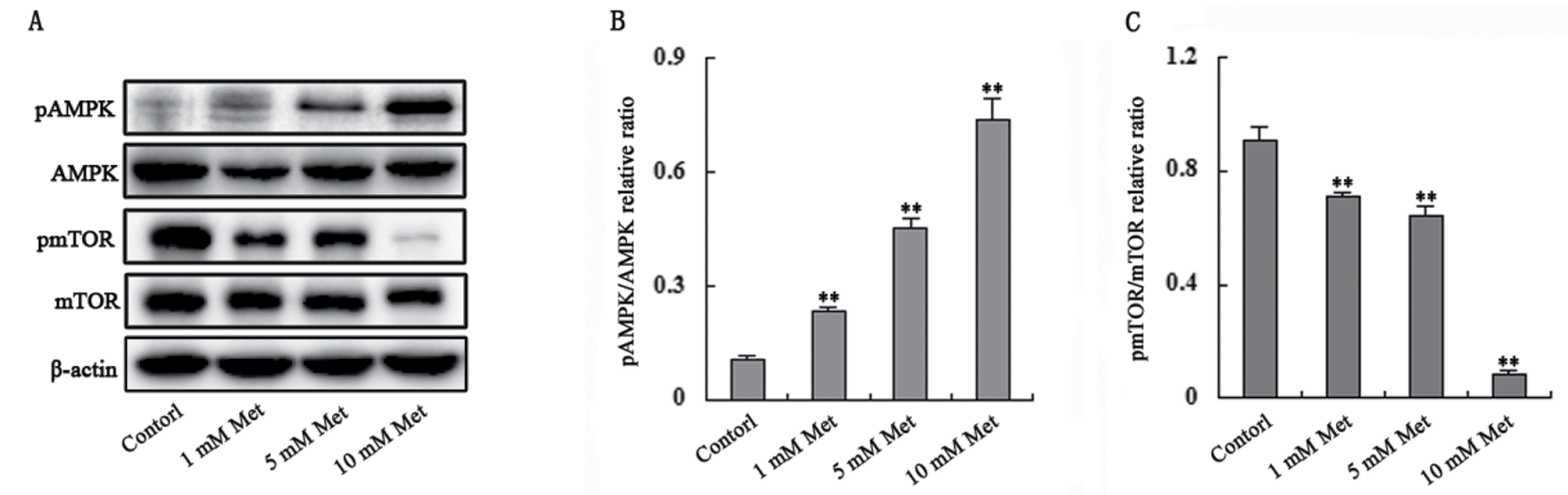
Met treatment increases phosphorylation of AMPK and decreases phosphorylation of mTOR. (A) Phosphorylation of AMPK was upregulated and phosphorylation of mTOR was downregulated in a dose-dependent manner. (B and C) Band intensities were quantified using Image Lab 5.0 software and were normalized to β-actin (**P<0.01, compared with the control). AMPK, AMP-activated protein kinase; mTOR, mammalian target of rapamycin; pAMPK, phosphorylated AMP-activated protein kinase; pmTOR, phosphorylated mammalian target of rapamycin; Met, metformin.

